# Investigation of the robustness of *Cupriavidus necator* engineered strains during fed-batch cultures

**DOI:** 10.1186/s13568-021-01307-4

**Published:** 2021-11-16

**Authors:** Catherine Boy, Julie Lesage, Sandrine Alfenore, Stéphane E. Guillouet, Nathalie Gorret

**Affiliations:** TBI, Université de Toulouse, CNRS, INRA, INSA, 135 Avenue de Rangueil, 31077 Toulouse Cedex 04, France

**Keywords:** Plasmid stabilization systems, Single cell analysis, Plasmid expression level, Cell permeability, Kinetics, Culture strategies

## Abstract

It is of major interest to ensure stable and performant microbial bioprocesses, therefore maintaining high strain robustness is one of the major future challenges in industrial microbiology. Strain robustness can be defined as the persistence of genotypic and/or phenotypic traits in a system. In this work, robustness of an engineered strain is defined as plasmid expression stability, cultivability, membrane integrity and macroscopic cell behavior and was assessed in response to implementations of sugar feeding strategies (pulses and continuous) and two plasmid stabilization systems (kanamycin resistance and Post-Segregational Killing *hok/sok*). Fed-batch bioreactor cultures, relevant mode to reach high cell densities and higher cell generation number, were implemented to investigate the robustness of *C. necator* engineered strains. Host cells bore a recombinant plasmid encoding for a plasmid expression level monitoring system, based on eGFP fluorescence quantified by flow cytometry. We first showed that well-controlled continuous feeding in comparison to a pulse-based feeding allowed a better carbon use for protein synthesis (avoiding organic acid excretion), a lower heterogeneity of the plasmid expression and a lower cell permeabilization. Moreover, the plasmid stabilization system Post-Segregational Killing *hok/sok*, an autonomous system independent on external addition of compounds, showed the best ability to maintain plasmid expression level stability insuring a greater population homogeneity in the culture. Therefore, in the case of engineered *C. necator*, the PSK system *hok/sok* appears to be a relevant and an efficient alternative to antibiotic resistance system for selection pressure, especially, in the case of bioprocess development for economic and environmental reasons.

## Keypoints

- Evaluation of engineered *Cupriavidus necator* stability and robustness via single-cell analysis
- Continuous feeding strategy less stressful than pulse-based feeding
- Higher cell homogeneity with *hok/sok* plasmid stabilization system than with kanamycin

## Introduction

Ensuring phenotypic homogeneity in engineered microorganisms is of major interest to enable maintaining production yields and avoiding process instability (Binder et al. 2017). However, the insertion of a recombinant plasmid generally leads to a metabolic load on host cells due to heterogeneous gene expression, plasmid maintenance and recombinant molecule production (Bentley and Quiroga 1993; Ceroni et al. 2018; Glick 1995; Lv et al. 2019; Million-Weaver and Camps 2014; Park et al. 2018; Silva et al. 2012). This means that two major biological mechanisms are competing within plasmid-bearing cells: plasmid maintenance and cell growth (Silva et al. 2012). It is generally admitted that plasmid-free cells grow faster than plasmid-bearing cells, resulting in a growth rate difference that intensifies segregational instability (Bentley et al. 1990; De Gelder et al. 2007; Glick 1995). Consequently, recombinant protein expression decreases globally and leads to the reduction of process performance.

Green Fluorescence Protein (*abbr*. GFP) and some of its derivatives such as enhanced GFP (*abbr.* eGFP), have been shown to be useful biosensors for the detection of variations in gene expression both in single-cells and in total populations (Argueta et al. 2004; Blokpoel et al. 2003; Cao and Kuipers 2018; Carroll et al. 2003; Chudakov et al. 2010; Morschhäuser et al. 1998; Utratna and O'Byrne 2014; Wons et al. 2018). In our previous work (Boy et al. 2020), a plasmid expression level monitoring method based on the expression of a plasmid-encoded eGFP biosensor has been designed. Our system was tested in culture conditions allowing validating its relevance to quantify both homogeneous and induced-heterogeneous cell populations. The results showed that this specific eGFP biosensor could be valuable to study both plasmid expression level variations under recombinant production of a molecule of interest in *Cupriavidus necator* and strain robustness under intensive production conditions. Strain robustness in that context corresponds to the capacity of the cells to keep their maximum performance during the bioprocess. This means that cells will retain their genetic background, in particular in the case of engineered cells overproducing product of interest, and will also maintain their fitness (membrane integrity, cultivability, and macroscopic behavior) in order to insure stable production.

*Cupriavidus necator* H16 is a chemolithoautotrophic bacterium, well-known for its capacity to produce and store up to 80% of its dry cell weight of Poly-β-hydroxybutyrate (*abbr.* PHB) (Pohlmann et al. 2006; Ryu et al. 1997). Its genome was entirely sequenced and mainly annotated (Cramm 2009; Pohlmann et al. 2006; Schwartz et al. 2003). *C. necator* has a versatile metabolism and is naturally able to consume organic carbon sources [fructose Budde et al. 2011; Grousseau et al. 2014), oils (Budde et al. 2011), formic acid (Grunwald et al. 2015), fatty acids (Johnson 1971; Wang et al. 2010), organic acids (Doi et al. 1988)] and inorganic ones [CO_2_ (Repaske and Mayer 1976; Tanaka et al. 1995)]. So, developing and improving tools for the genetic engineering of *C. necator* would open up to new possibilities in terms of synthetic biology of the strain. The interest in developing a complete genetic toolbox for *C. necator* has intensified in recent years, especially for plasmid construction (Bi et al. 2013; Gruber et al. 2014; Sato et al. 2013; Sydow et al. 2017). To increase strain robustness, examples of stabilizing mechanisms have already been efficiently transposed to recombinant plasmids in *C. necator* through stabilizing cassette insertion. They are classified in three main stabilizing categories. First, plasmid addiction systems consist in the killing of plasmid-free cells, or the reduction of their growth rate (Friehs 2004). Three of them have already been adapted to *C. necator*: antibiotic resistance (*e.g.* kanamycin (Grousseau et al. 2014; Gruber et al. 2014), chloramphenicol (Sydow et al. 2017)), chromosomal mutation complementation (*e.g.* single-cell auxotrophy through KDPG-aldolase (Voss and Steinbuchel 2006), proline (Budde et al. 2011)) and Post-Segregational Killing (*e.g. parDE* operon from the RP4 plasmid (Gruber et al. 2014)). Second, site-specific recombination systems ensure that plasmid multimers formed during replication and/or recombination can be resolved by a site-specific recombination system. Each monomer is transmitted independently to daughter cells. This system can also be referred as plasmid multimer resolution system (Zielenkiewicz and Ceglowski 2001). The *parCBA* operon encoding for the multimer resolution system of the plasmid RK2 (or RP4) (Easter et al. 1998) from *Escherichia coli* has been tested in *C. necator* (Gruber et al. 2014). Third, active partitioning systems ensure that the plasmid copies are vertically transmitted efficiently to every daughter cell (Million-Weaver and Camps 2014; Schwartz et al. 2003; Zielenkiewicz and Ceglowski 2001). The partition locus of the megaplasmid pMOL28 from *C. metallidurans* CH34 has been successfully applied in *C. necator* H16 (Sato et al. 2013).

Hereinafter, attention was drawn to two plasmid addiction systems: kanamycin resistance and *hok/sok* Post-Segregational Killing system. On one hand, kanamycin resistance is rather commonly used with plasmids of.

*C. necator* (Grousseau et al. 2014; Gruber et al. 2014; Marc et al. 2017). On the other hand, to our knowledge, the *hok/sok* PSK system has never been used in *C. necator* before. First, the mode of action of kanamycin consists in interfering with protein synthesis by binding to bacterial ribosome. This leads to an incorrect alignment with mRNA and consequently to an amino acid misreading during protein synthesis. Non-functional peptide chains are synthesized. The kanamycin resistance system used here is neomycin phosphotransferase II (*abbr.* NPTII) from the *neo* gene (*i.e.* neomycin-resistance) of the transposon Tn5, which is part of the aminoglycoside.

3’-phosphotransferase APH (3’)-II subclass (Yenofsky et al. 1990). Its mode of action consists in catalyzing the ATP-dependent phosphorylation of kanamycin on its 3’-hydroxyl group and thus, making the antibiotic chemically unstable (Haas and Dowding 1975; Ramirez and Tolmasky 2010; Wright 1999; Yenofsky et al. 1990). Second, the Post-Segregational Killing system *hok/sok* from the R1 plasmid of *E. coli* ensures plasmid stabilization by the killing of plasmid-free cells. It encodes for two RNA: *hok* mRNA and *sok* antisense RNA. The *hok* gene encodes for the toxin protein Hok. The *sok* antisense RNA indirectly regulates *hok* translation (Thisted et al. 1994). Plasmid stabilization mechanism of the PSK system *hok/sok* is based on the differential decay rate between *sok* antisense RNA, who has the highest, and the Hok toxin (Thisted et al. 1994). During cell division, if the plasmid is transmitted only to one of the two daughter cells, plasmid-bearing cells will express the *sok* antisense RNA to block Hok toxin actions and remain viable. Plasmid-free cells die from the toxin because the unstable antisense *sok* RNA antitoxin has been degraded (Cooper and Heinemann 2000; Friehs 2004).

In addition, fluctuating environments are known to generate and/or amplify population heterogeneities and a loss of strain robustness (Barkai and Shilo 2007; Delvigne et al. 2015; Limberg et al. 2017; Masel and Siegal 2009). In Nature, cell populations have to adapt to fluctuating growth conditions (*e.g.* temperature, pH, nutrients, toxin concentrations). To do so, cell populations may improve their fitness thanks to individuals that evolve stochastically between several different phenotypes. Therefore, some cells might always be prepared to face sudden environmental fluctuations (Acar et al. 2008; Kussell and Leibler 2005). In industrial bioprocesses, the apparition of such transitory fluctuations (*e.g.* temperature, pH, nutrient concentrations) might greatly disrupt process performances: reduction of production and biomass yield, decreased growth and production rates (Hewitt et al. 2007; Lara et al. 2006; Limberg et al. 2017).

The objective of this work was to compare the robustness (as previously defined as plasmid stability, membrane integrity, cultivability and macroscopic microbial behavior) of *C. necator* strains carrying different plasmid stabilization systems (Kanamycin resistance and Post Segregational Killing systems) under well-controlled culture condition similar to the one optimized for recombinant overproduction of product of interest such as isopropanol (Grousseau et al. 2014; Marc et al. 2017). Two fermentation strategies were applied, pulse-based fed-batch or continuous feeding fed-batch, and evaluated. More specifically, the impact of plasmid expression levels of the recombinant protein was investigated through eGFP single-cell fluorescence.

## Materials and methods

### Strain, plasmids and media

#### Strains

The expression strain used in this study was *Cupriavidus necator* Re2133 (Budde et al. 2011). This strain was derived from the wildtype strain *C. necator* H16/ATCC17699, whose PHB production pathway was deleted (acetoacetyl-CoA reductases, *phaB1B2B3*; PHA synthase, *phaC1*). *Cupriavidus necator* Re2133 was gentamicin resistant (Gen^R^). Plasmid constructions were achieved through the strains *Escherichia coli* S17-1 and Top10.

#### Plasmids

The plasmids pCB1 and pCB3 were used in this work. The design and associated molecular biology protocols for the construction of the plasmid pCB1 were explained in more details in Boy et al. (2020). The plasmid pCB3 was constructed following the same methodology, from the plasmid backbone pBBAD-Par. The plasmid pCB3 encodes for the Post-Segregational Killing (*abbr.* PSK) system *hok/sok*. Both plasmids encode for the *P*_*lac*_*-eGFP* cassette and for kanamycin resistance (Kan^R^).

#### Media

The rich media used for precultures were Tryptic Soy Broth (TSB, Becton Dickinson, Sparks, MD, USA) for liquid cultures and Tryptic Soy Agar (*abbr.* TSA, TSB + 20 g·L^−1^ agar) for Petri dishes. Gentamicin (10 mg·L^−1^) and kanamycin (200 mg·L^−1^) were added in theses media. Lysogeny broth medium (*abbr.* LB) was used for molecular biology as described in Boy et al. (2020).

The mineral medium used for flasks cultivation was previously described in Lu et *al.* (Lu et al. 2013) and Boy et al. (2020). To maintain selection pressure, gentamicin (10 mg·L^−1^) and kanamycin (200 mg·L^−1^) were added in the mineral medium.

The mineral medium used for bioreactor cultivation was composed as follows (per liter): (NH_4_)_2_SO_4_, 2.8 g; MgSO_4_-7H_2_O, 0.75 g; phosphorus (Na_2_HPO_4_-12H_2_O, 1.5 g; KH_2_PO_4_, 0.25 g); nitrilotriacetic acid, 0.285 g; ammonium iron(III) citrate (28%), 0.09 g; CaCl_2_, 0.015 g; trace elements (H_3_BO_3_, 0.45 mg; CoCl_2_-6H_2_O, 0.3 mg; ZnSO_4_,7H_2_O, 0.15 mg; MnCl_2_-4H_2_O, 0.045 mg; Na_2_MoO4-2H_2_O, 0.045 mg; NiCl_2_-6H_2_O, 0.03 mg; CuSO_4_, 0.015 mg). Fructose was used as sole carbon source with an initial concentration of 50 g·L^−1^ for the culture with pulses and 30 g·L^−1^ for the culture with continuous feeding. For experiments containing kanamycin as selection pressure, 100 mg·L^−1^ of kanamycin was added in the medium at inoculation and every 10 g_CDW_·L^−1^ of biomass produced.

### Precultures on fructose

Precultures were achieved as previously described in Boy et al. (2020).

### Fed-batch cultivations on fructose

Batch cultivations consisted in non-limited growth on fructose at 30 °C and pH7. The set-up (regulation, monitoring) described in Boy et al. (2020) was used.

Fed-batch phases were initiated when nitrogen exhaustion was reached in the bioreactor mineral medium (*i.e.* corresponding to 5 g_CDW_·L^−1^ biomass). Nitrogen (ammonium, NH_4_^+^) was the limiting substrate and was fed in the bioreactor through calibrated peristaltic pumps with an exponential flow rate set at 0.04 h^−1^. This growth rate was chosen based on previous works to investigate isopropanol producing conditions, with fructose pulse feeding strategy (Marc et al. 2017). Initial fructose concentration was equal to 50 g·L^−1^ for the culture with pulses and 30 g·L^−1^ for the culture with continuous feeding.

In that specific work, two fedbatch strategies were carried out for fructose feeding in order to evaluate the impact of pulses versus smooth continuous feeding:

1. **Fructose pulse strategy**: when fructose concentration reached 20 g·L^−1^ in the bioreactor, a pulse was performed to reach 50 g·L^−1^ of fructose.
2. **Controlled feeding strategy**: when fructose concentration reached 20 g·L^−1^, fructose was fed exponentially in the bioreactor to maintain a constant residual concentration of 20 g·L^−1^.

To prevent nutrient limitation, a phosphorus solution (7 mL·L^−1^) and a trace elements solution (2 mL·L^−1^) were added every 10 g_CDW_·L^−1^ of biomass produced. Plus, to maintain selection pressure, a kanamycin solution at 50 g·L^−1^ was also added (2 mL·L^−1^) every 10 g_CDW_·L^−1^.

### Analytical procedures

#### Biomass characterization

Biomass concentration was measured by optical density (OD) at 600 nm using a visible spectrophotometer (DR3900, Hachlange, Loveland, Colorado, USA) with a 0.2 cm path length absorption cell (Hellma). OD was correlated to cell dry weight (CDW) measurements (*i.e.* 2 g_CDW_·L^−1^ = 1 OD unit), as described in Boy et al. (2020). The results of biomass determinations were reproducible within 5% in replicate assays.

#### Metabolite quantification

Cells samples were centrifuged, and supernatants were filtrated (0.2 μm PTFE syringe filters, VWR) before being used for substrate and products determination. The residual fructose and organic acids concentrations were quantified by high-performance liquid chromatography (HPLC). The HPLC instrument (Series 1100, Agilent) was equipped with an ion-exchange column (Aminex HPX-87H, 300 × 7.8 mm, Bio-Rad, Hercules, CA, USA) protected with a guard column (Cation H^+^ cartridge, 30 × 4.6 mm, Bio-Rad) and coupled to a RI detector and an UV detector (λ = 210 nm). The column was eluted with 2.5 mM H_2_SO_4_ as a mobile phase at 50 °C at a flow rate of.

0.5 mL·min^−1^. Residual nitrogen was quantified by higher-pressure ionic chromatography (HPIC). The HPIC instrument (ICS-2100 RFIC, Dionex) was equipped with an IonPac™ CS16 column (RFIC™, 3 × 50 mm, BioRad) and an ion suppressor CERS 500 (2 mm, Thermo Scientific). The column was eluted with 30 mM metanesulfonic acid as a mobile phase at 40 °C and a 40 mA ion suppressor current, at a flow rate of 0.36 mL·min^−1^. The systematic error in the quantification of metabolites was determined to be less than 5% from replicates.

#### Plate count

Plasmid stability was quantified by parallel plate count on antibiotic selective TSB Petri dishes (10 mg·L^−1^ Gentamicin and 10 mg·L^−1^ Gentamicin + 200 mg·L^−1^ Kanamycin). Serial dilutions were performed in physiological water (0.85% NaCl) tubes (BioMérieux, Marcy-l’Étoile, France). For every sample, three dilutions were tested, between 10^–5^ and 10^–9^. The diluted sample were plated in triplicate with the Whitley Automated Spiral Plater (Don Whitley Scientific, Shipley, UK). Decimal reduction rate from plate count measurements was calculated as: $N = log\left( {\frac{{Gen^{R} cells}}{{Gen^{R} Kan^{r} cells}}} \right)$.

#### Flow cytometry

The BD Accuri C6® flow cytometer (BD Biosciences, Franklin Lakes, NJ, USA) was used to measure cell permeability to propidium iodide (FL3 channel) and eGFP-fluorescence of plasmid-expressing cells (FL1 channel). Cell samples were diluted in physiological solution at 10^6^ cells·mL^−1^ and then, were stained with 20 µL of a commercial solution of propidium iodide at 20 mg L^−1^ (*abbr.* PI) (Molecular Probes, Invitrogen, USA) and incubated 20 min at room temperature in the dark. A 100% dead–cell control was prepared by incubating cells in 70% isopropanol for 1 h at room temperature. Samples were run until 20, 000 events were counted at 14 μL·min^−1^ using milli-Q water as sheath fluid. The Forward Scatter Signal (threshold: 12, 000) and Side Scatter Signal (threshold: 2, 000) were used as trigger channels. Data acquisition was performed with BD Accuri CFlow® software and data processing was achieved with FlowJo software (Becton Dickinson, Sparks, MD, USA). Decimal reduction rate was calculated as described above from plasmid-expressing cells (eGFP-positive cells, FL1-A > 8·10^2^) and total cells (Single cells, bisectors of both FCS-A *vs* FSC-H and SSC-A *vs* SSC-H): $N = log\left( {\frac{Single - cells}{{eGFP - cells}}} \right)$.

#### Extracellular fluorescence measurement

Extracellular fluorescence measurements were achieved as described in Boy et al. (2020). Relative extracellular fluorescence intensity was calculated as: $RFU = \frac{{FU_{t} - FU_{{t_{0} }} }}{{OD_{t} - OD_{{t_{0} }} }}$.

### Data analysis

Specific substrate consumption (fructose $q_{S}$, ammonium $q_{N}$) and biomass production ($\mu$) rates were calculated from experimental data and mass balances (carbon, nitrogen and elemental). Specific growth rate was determined as $\ln \left( {X} \right) = f\left( t \right)$ and its error was calculated as the standard deviation of the slope. Determination of specific oxygen consumption ($q_{{O_{2} }}$) and carbon dioxide production ($q_{{CO_{2} }}$) was based on mass balance calculations in both liquid and gaseous phase, from inlet/outlet gas composition, temperature, pH, stirring, oxygen partial pressure (pO_2_), and liquid volume. For overall production/consumption yield calculation, masses were plotted pairwise in a scatter plot. A linear regression was used to determine the considered yields and the error was calculated by the standard deviation of the slope.

#### Statistical analysis: normality of distribution functions by BoxPlot representation

Boxplots represented distributions through graphical localization parameters, such as the median (50th percentile), the first (25th percentile) and third quartiles (75th percentile). The first and third quartiles represented the bottom and top of the boxplot, respectively. The line inside the box symbolized to the median. The interquartile range (*abbr.* IQR) corresponded to the length of the boxplot and was situated between the first and third quartiles. The whiskers symbolized the minimum and maximum values, when comprised within 1.5 × IQR from both extremities. Outliers, above 1.5 × IQR, were represented by points. A boxplot symmetrically centered on the median might be expected to be normally distributed (Rakotomalala 2011).

## Results

### Impact of fructose feeding strategy

In order to evaluate whether fluctuating nutrient environment may impact the strain robustness (defined as plasmid expression stability, cultivability, membrane integrity and macroscopic cell behavior of engineered *C. necator* strains), two different sugar feeding strategies were carried out. Nitrogen-limited fed-batch cultures were performed by implementing either a pulse-based fructose feeding or a continuous fructose feeding. The plasmid monitoring system developed in Boy et al. (2020), based on the expression of eGFP by plasmid-bearing cells was used. The pCB1 strain was grown under selection pressure (*i.e.* 100 mg·L^−1^ kanamycin addition at inoculation and every 10 g_CDW_·L^−1^ of biomass produced).

#### Growth kinetics characteristics

**Fig. 1:**
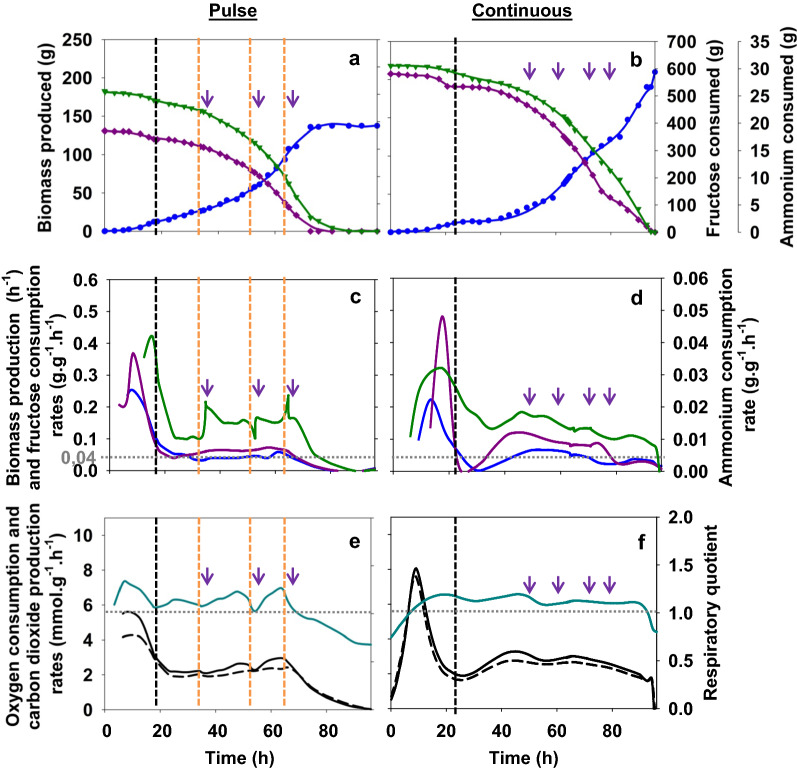
Cumulated biomass production ( ), fructose ( ) and ammonium ( ) consumption during culture of the strain Re2133/pCB1 with kanamycin: with fructose pulses (**a**) and continuous feeding (**b**). Growth rate ( ), fructose ( ) and ammonium ( ) consumption rates during Re2133/pCB1 culture of the strain Re2133/pCB1 with kanamycin: with fructose pulse (**c**) and continuous feeding (**d**). Respiratory quotient ( ), oxygen consumption ( ) and carbon dioxide ( ) production rates during culture of the strain Re2133/pCB1 with kanamycin: with fructose pulse (**e**) and continuous feeding (**f**). Black vertical lines represent the beginning of the fed-batch phase and orange vertical lines represent fructose pulses. Purple arrows represent additions (kanamycin, 100 mg·L^−1^; elements and phosphorus solutions)

**Fig. 2:**
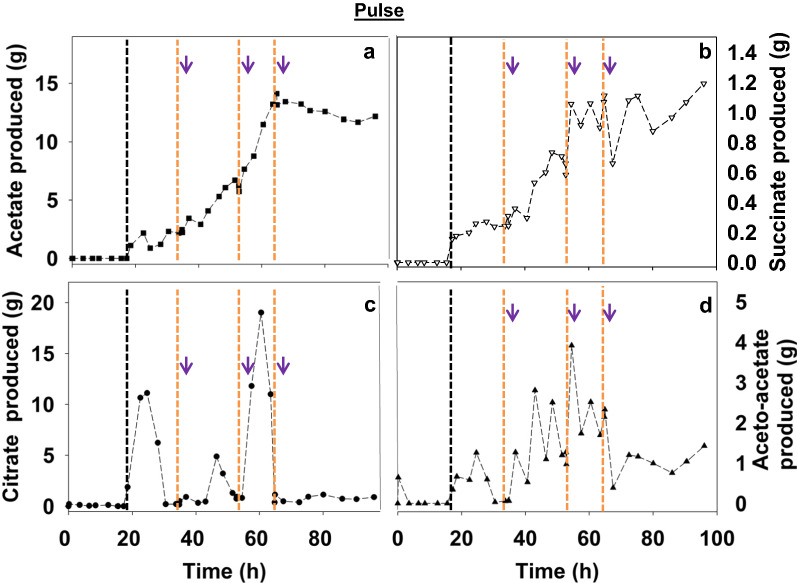
Organic acids production during fructose pulses: acetate (**a**: ■), succinate (**b**: ▽), citrate (**c**: ●), and aceto-acetate (**d**: ▲). Black vertical lines represent the beginning of the fed-batch phase and orange vertical lines represent fructose pulses. Purple arrows represent additions (kanamycin, 100 mg·L^−1^; elements and phosphorus solutions)

Carbon, nitrogen and elemental balances were checked and completed at least at 90% for both cultures. A final biomass production (Fig. 1a, b) of 137 g for pulses and 210 g for continuous feeding was obtained. The global biomass production yields from fructose were equal to 0.28 ± 0.01 g_X_·g_S_^−1^ and 0.32 ± 0.01 g_X_·g_S_^−1^ for pulse and continuous feeding, respectively (Table 1). No organic acid, other than pyruvate, was produced during culture with continuous fructose feeding. A slight transient peak of pyruvate was detected after the initiation of the fed-bath phase once nitrogen was exhausted for both conditions, at 4.96 g for continuous feeding and 4.86 g for pulses. However, other organic acids were produced only during the culture with fructose pulses (Fig. 2): citrate, acetate, aceto-acetate and succinate to a lesser extent. This production represented 15% of the carbon flow during fed-batch culture. For acetate and succinate, the mass produced increased continuously from the beginning of the fed-batch phase; but, for acetate only until biomass started slowing down. For aceto-acetate, production was very noisy and started at the beginning of the fed-batch phase. For citrate, three peaks of production could be observed, after the beginning of the nitrogen–limited phase and after the first two fructose pulses. However, it was re-consumed after each pulse and represented less than 1% of the carbon flow.

**Table 1:** Summary of macroscopic data for the strains Re2133/pCB1 (with or without kanamycin; with pulse or continuous fructose feeding) and pCB3

Strains	$\mu$	R_S/X_	R_NH3/X_	R_S/CO2_	By-products	RQ	q_CO2_	q_O2_	
	(h^−1^)	(g_X_·g_S_^−1^)	(g_X_·g_NH3_^−1^)	(g_CO2_·g_S_^−1^)			(mmol·g_X_^−1^·h^−1^)	(mmol·g_X_^−1^·h^−1^)	
Re2133/pCB1 + Kan *Pulse feeding*	0.25 ± 0.01	0.28 ± 0.01	6.6 ± 0.2	0.74 ± 0.01	acetate, succinate, aceto-acetate, citrate	1.12 ± 0.08	2.41 ± 0.30	2.14 ± 0.22	
Re2133/pCB1 + Kan *Continuous feeding*	0.22 ± 0.01	0.32 ± 0.01	5.4 ± 0.2	0.85 ± 0.02	none	1.13 ± 0.03	2.81 ± 0.57	2.48 ± 0.49	
Re2133/pCB1 *Continuous feeding*	0.24 ± 0.01	0.32 ± 0.01	5.60 ± 0.08	0.89 ± 0.02	none	1.05 ± 0.06	2.14 ± 0.23	2.05 ± 0.24	
Re2133/pCB3 *Continuous feeding*	0.20 ± 0.01	0.32 ± 0.01	5.21 ± 0.41	0.87 ± 0.02	none	1.14 ± 0.02	2.10 ± 0.40	1.84 ± 0.30	
Reference (Aragao et al. 1996)		0.53	5.96	0.51

During batch phase, the specific growth rates for both conditions were equal to 0.25 ± 0.01 h^−1^ and.

0.22 ± 0.01 h^−1^, respectively with the initial fructose concentration at 30 and 50 g·L^−1^ (Fig. 1c, d). Then, growth dynamics were imposed by the nitrogen feeding rates around 0.04 h^−1^ for both culture condition during fed-batch phase. The number of cell generations produced was 7 with fructose pulse and 8 with continuous feeding. With fructose pulses, growth slowed down after 65 h, just after the third fructose pulse, and stopped at 7.25 generations (80 h) (Fig. 1a, c). During fed-batch phase for continuous feeding, fructose consumption rate was close to 0.14 ± 0.03 g_S_·g_X_^−1^·h^−1^. For pulse feeding, fructose consumption rate increased in response to every fructose addition and then stabilized around 0.15 g_S_·g_X_^−1^·h^−1^ in-between pulses. Meanwhile, ammonium consumption rate was close to 0.006 ± 0.001 g_NH3_·g_X_^−1^·h^−1^ with fructose pulse and to 0.008 ± 0.004 g_NH3_·g_X_^−1^·h^−1^ for continuous feeding (Fig. 1c, d). During the pulse experiment, ammonium consumption rate dropped just after the third fructose pulse (Fig. 1c).

The respiratory quotient (*abbr.* RQ) was stable around 1.13 ± 0.03 during fed-batch phase (20—90 h) for continuous feeding (Fig. 1f; Table 1). Specific CO_2_ production and O_2_ consumption rates were comprised around.

2.81 ± 0.57 and 2.48 ± 0.49 mmol·g^−1^·h^−1^, respectively. For fructose pulses during fed-batch, respiratory quotient seemed to transiently decrease in response to fructose pulses, until the third fructose pulse where it constantly decreased to 0.7 (Fig. 1e; Table 1). On this time period, such a decrease in the RQ might be explained by the production of organic acids due to their more oxidized nature (*i.e.* degrees of reduction of 3.0 for citric and acetoacetic acids, 3.33 for pyruvic and 3.5 for succinic compared to 4.2 for biomass) when growth had stopped (theoretical RQ at 1.06 for growth only). CO_2_ specific production rate and O_2_ specific consumption rate were comprised around 2.41 ± 0.30 and 2.14 ± 0.22 mmol·g^−1^·h^−1^, respectively; a slight increase in carbon dioxide production rate was detected after the two first pulses.

#### Single-cell analysis of plasmid expression levels

Single-cell analysis was supported by flow cytometry data, and plasmid expression levels analysis by both plate count and flow cytometry measurements. All these data were studied in light of physiological measurements (*i.e.* cell permeability measured by PI staining).

**Fig. 3:**
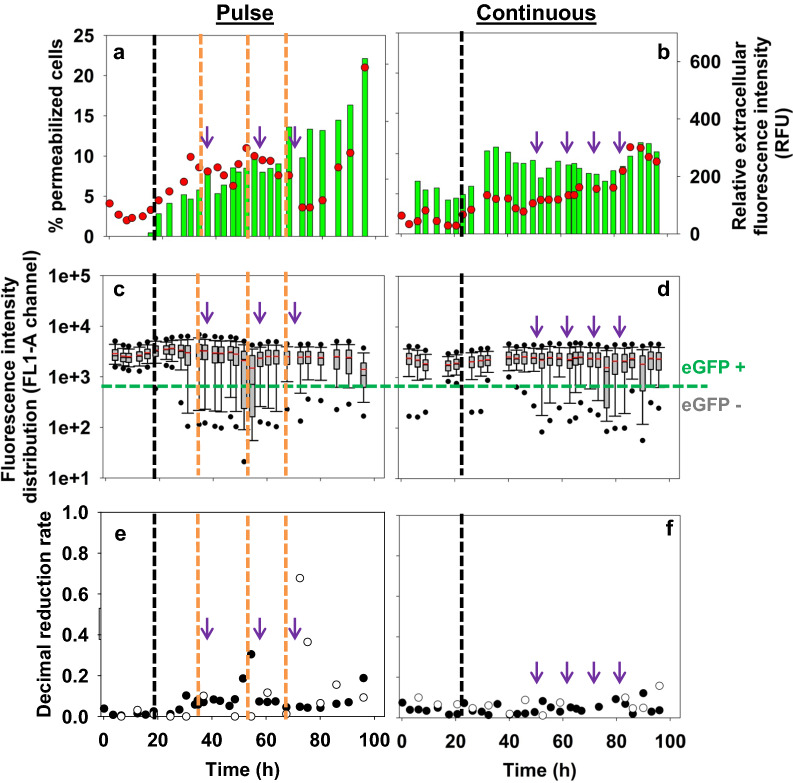
Relative extracellular fluorescence intensity (FU/OD_intact_) ( ) and percentage of permeabilized cells ( ) in the FL3-A channel for Re2133/pCB1 with kanamycin: with fructose pulse (**a**) and continuous feeding (**b**). Boxplot comparison on fluorescence intensity distribution in the FL1-A channel for Re2133/pCB1 with kanamycin: with fructose pulse (**c**) and continuous feeding (**d**). Evolution of the decimal reduction rate for Re2133/pCB1 with kanamycin: with fructose pulses (**e**) and continuous feeding (**f**) based on plate count (□) and flow cytometry (●) measurements. Black vertical lines represent the beginning of the fed-batch phase and orange vertical lines represent fructose pulses. Purple arrows represent additions (kanamycin, 100 mg·L^−1^; elements and phosphorus solutions)

During batch phase, the cell permeability percentage (Fig. 3a, b) was low (below 5%) for both conducts. Meanwhile, relative extracellular fluorescence intensities were weak between 100–200 RFU, in all tested conditions. An increase was observed at nitrogen depletion for both experiments (6 and 8%). In fed-batch phase, the percentage of permeabilized cells was overall higher when driving by pulse (10–20%) than by continuous feeding (5–10%). Furthermore, a clear difference was observed after the third addition of fructose when the percentage of permeabilized cells rose to 20% while it remained below 10% with continuously feeding. Thus, linked to permeability increase, the relative extracellular fluorescence intensity was higher in the pulse mode and increased all along the culture, up to 619 RFU. In the continuously fed reactor, relative extracellular fluorescence intensity was kept more stable compared to fructose pulses, as the percentage of permeabilized cells remained closer to a constant, suggesting equilibrium between new formed cells by growth and permeabilized ones.

Boxplots representing single-cell fluorescence intensity distribution within the population were globally wider in the pulse mode during fructose addition (Fig. 3c), especially in the direction of the first quartile. The fluorescence intensity distribution was close to normal (*i.e.* median = mean and first quartile length = third quartile length) for continuous fructose feeding (until 50 h, Fig. 3d) and during pulses (until 30 h, Fig. 3c); even if there was a slight heterogeneity at the outliers (*i.e.* extreme values, 5 and 95% of the distribution) in continuous feeding (Fig. 3d). After that, distribution range increased for both feeding strategies. After 60 h of culture, boxplots presented a wider and non-Gaussian distribution range for all culture conditions. Distribution ranges were noisier for continuous feeding compared to pulse feeding when growth had stopped.

With pulse feeding, the decimal reduction rates (Fig. 3e) increased significantly at 55 h (by flow cytometry) and 70 h (by plate count), respectively after the second and third fructose pulses. With continuous fructose feeding (Fig. 3f), decimal reduction rates (for flow cytometry and plate count) were low and not significant (< 0.2) throughout the culture.

The continuous fructose feeding strategy allowed both slightly more stable plasmid expression levels and a significant more efficient macroscopic behavior in terms of cell permeability and overall production yields, compared to pulse fructose feeding. Thus, the continuous strategy was retained for the fed-batch cultures in order to investigate the different plasmid stabilization systems.

#### Impact of plasmid stabilization systems

In order to assess the efficiency of plasmid stabilization systems in maintaining strain robustness, fed-batch experiments were carried out with strains expressing two different plasmid stabilization systems. Kanamycin resistance (strain Re2133/pCB1) and Post-Segregational Killing system *hok/sok* (strain Re2133/pCB3) were evaluated based on their contribution to strain robustness: macroscopic, physiological behavior and expression stability. Fed-batch experiments were carried out following the strategy described above with continuous fructose feeding.

#### Systems based on plasmid-encoded antibiotic resistance

The plasmid stabilization system based on kanamycin resistance was expressed in a constitutive manner on the strain Re2133/pCB1. Fed-batch cultures with and without kanamycin selection pressure were implemented. Kanamycin (100 mg·L^−1^) was added at inoculation and every 10 g_CDW_·L^−1^ of biomass produced.

#### Growth kinetics characteristics

**Fig. 4:**
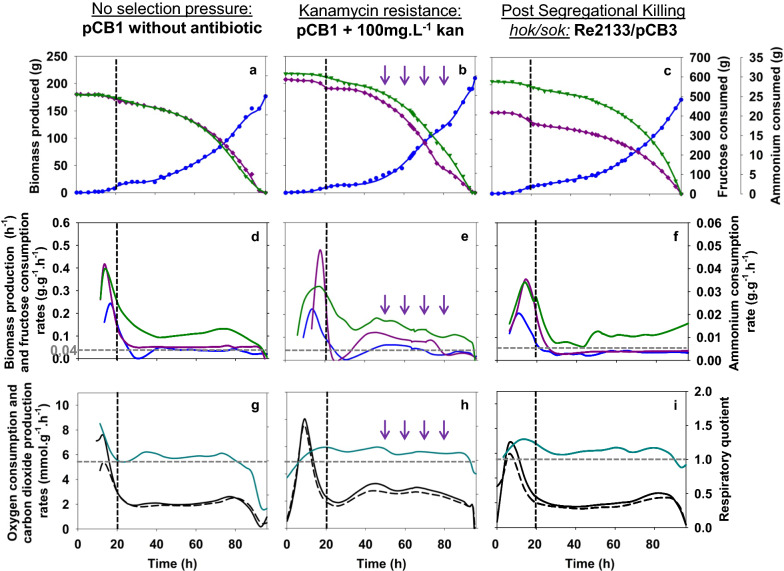
Biomass production ( ), fructose ( ) and ammonium ( ) consumption during culture of the strains Re2133/pCB1 (without (**a**) and with kanamycin (**b**) and pCB3 (**c**)). Growth rate ( ), fructose ( ) and ammonium ( ) consumption rates during Re2133/pCB1 culture of the strains Re2133/pCB1 (without (**d**) and with kanamycin (**e**)) and pCB3 (**f**)). Respiratory quotient ( ), oxygen consumption ( ) and carbon dioxide ( ) production rates during culture of the strains Re2133/pCB1 (without (**g**) and with kanamycin (**h**) and pCB3 (**i**)). Black vertical lines represent the beginning of the fed-batch phase and purple arrows represent additions (kanamycin, 100 mg·L^−1^; elements and phosphorus solutions)

For both fermentation conditions, carbon, nitrogen and elemental balances closed above 95%. Final biomass production reached for both strains was 178 g without antibiotic and 210 g with kanamycin after 8 cell generations (Fig. 4a, b). In order to evaluate differences in terms of carbon, nitrogen and oxygen repartition according to the plasmid management, overall yields were calculated and compared to the theoretical values. There was no difference in the values of overall yields measured between the batch phase and the fed-batch phase in both culture conditions. Both overall growth yields were equal to 0.32 ± 0.01 g_X_·g_S_^−1^, a 40% decrease compared to the theoretical biomass production yield from fructose (0.53 g_X_·g_S_^−1^ (Aragao 1996)) (Table 1). This decrease in biomass overall yield from fructose was due to diversion of the carbon flow toward CO_2_ production in our culture conditions. Its overall yield from fructose was always comprised in the same order of magnitude at 0.89 ± 0.02 g_CO2_·g_S_^−1^ and 0.85 ± 0.02 for g_CO2_·g_S_^−1^ without and with kanamycin, respectively (Table 1). No organic acids were produced during both cultures, except pyruvate produced transiently after the beginning of the fed-batch phase when nitrogen was exhausted then quickly consumed. For nitrogen, overall biomass production from ammonium was equal to 5.60 ± 0.08 g_X_·g_NH3_^−1^ without antibiotic and 5.40 ± 0.20 g_X_·g_NH3_^−1^ with kanamycin (Table 1). This difference appears non-significant based on the value of the standard deviation between the two culture conditions. Nevertheless, these yields were 6 to 9% lower than the theoretical one (5.96 g_X_·g_NH3_^−1^).

During batch phase (Figs. 4d, e), nitrogen limitation occurred when the biomass concentration reached 4 g_CDW_·L^−1^. For the strain *C. necator* Re2133/pCB1, the specific growth rate reached 0.22 ± 0.01 h^−1^ and 0.24 ± 0.01 h^−1^, with and without kanamycin respectively. During fed-batch phase, growth dynamics were imposed by the nitrogen feeding rates around 0.04 h^−1^ for both culture conditions. Specific consumption rates of fructose and ammonium during fed-batch phase were overall slightly higher with kanamycin addition; but, decreased all along fed-batch phase.

The respiratory quotient was stable around 1.05 ± 0.06 during fed-batch phase (20—90 h) without antibiotic (Fig. 4g; Table 1). Meanwhile, the respiratory quotient with kanamycin was stable around 1.13 ± 0.03 during fed-batch phase (20—90 h). (Fig. 4h; Table 1).

#### Single cell analysis

**Fig. 5:**
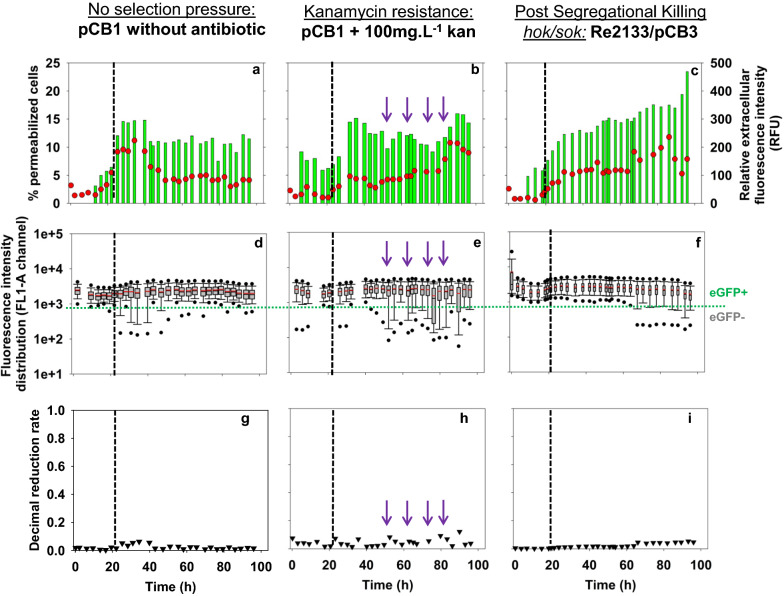
Relative extracellular fluorescence intensity (FU/OD) ( ) and percentage of permeabilized cells ( ) in the FL3-A channel for Re2133/pCB1 without (**a**) and with (**b**) kanamycin and pCB3 (**c**). Boxplot comparison on fluorescence intensity distribution in the FL1-A channel for Re2133/pCB1 without (**d**) and with (**e**) kanamycin and pCB3 (**f**). Evolution of the decimal reduction rate N (▼) during pCB1 without (**g**) and with (**h**) kanamycin and pCB3 (**i**). Black horizontal lines represent the beginning of the fed-batch phase. Ppurple arrows represent additions (kanamycin, 100 mg·L^−1^; elements and phosphorus solutions)

In all culture conditions, an increase in the permeabilized cells percentage was observed just after the beginning of the fed-batch phases; slight with kanamycin addition (2 to 5%) and more important without it (2 to 10%) (Fig. 5a, b). This could be explained as a direct answer to the transient nitrogen depletion that cells face at the end of the batch phase. For the strain Re2133/pCB1 without kanamycin, the value of permeabilized cells was higher, because a slightly longer starvation phase (*i.e.* 3 h instead of less than 1 h) before nitrogen was fed exponentially in the medium. At the end of the fed-batch phase, the maximum percentage of permeabilized cells was higher in presence of kanamycin, with 15% instead of 5% without selection pressure, both at 170 g of biomass produced. Relative extracellular fluorescence intensity increased mainly after the beginning of the fed-batch phase for both culture conditions and remained quite constant along the cultures. During fed-batch phase, its value was close to 175 RFU and 200 RFU with and without kanamycin addition, respectively and its evolution roughly followed the curve of the percentage of permeabilized cells for both culture conditions.

Intracellular eGFP distribution was represented by boxplots to highlight fluorescence intensity distribution at the single-cell level throughout fermentation. Boxplot distribution was close to normal during most of the batch phase for both culture conditions (Fig. 5d, e). There was a slight heterogeneity at the level of the outliers (*i.e.* extreme values, under 5 and above 95% of the total distribution) for kanamycin addition (Fig. 5e). After the beginning of the fed-batch phase, boxplot distribution range increased. Without antibiotic, the distribution range returned to a normal configuration after 55 h and until the end of the culture. With kanamycin, the boxplot distribution range was close to normal until 50 h, even if extreme values were rather low. After that timepoint, the distribution profile increased and boxplots were not normal anymore.

To evaluate plasmid expression loss, decimal reduction rate was calculated from two counting methods: traditional plate count method based on expression of kanamycin resistance encoded on the plasmid, and eGFP biosensor monitoring method (Fig. 5g, h). Only data from flow cytometry were shown here, as they were similar to results from plate count. Decimal reduction rate was low for all strains and culture conditions tested, and plasmid stability loss was very slight.

So, the widening in plasmid expression level distribution might be due to two phenomena: (1) the increase in the percentage of permeabilized cells and eGFP leakage (*i.e.* PI-positive cells; either eGFP-positive or -negative) compared to the non-selective condition, and (2) a very slight plasmid expression loss (*i.e.* eGFP-negative and PI-negative cells).

Plasmid stabilization system based on kanamycin resistance (with kanamycin addition) was accompanied by an higher heterogeneity in the plasmid expression level, as seen through boxplots, and induced a slight increase of permeabilization percentage of cells after 80 h. Nevertheless, plasmid stability was maintained during 8 cells generations even without addition of selection pressure.

#### System based on plasmid-encoded Post Segregational Killing System

The second plasmid stabilization system tested was based on the expression of a toxin/anti toxin system (*hok/sok*) which was expressed in a constitutive manner on the pCB3 plasmid.

#### Growth kinetics characteristics

Carbon, nitrogen and elemental balances were recovered (> 95%). Final biomass production reached for pCB3 with 173 g over 8.16 generations was close to those obtained for pCB1 without kanamycin addition, considered as reference culture (Fig. 4a, c; Table 1). Like stated beforehand, there was no difference between the values of overall yields measured between the batch phase and the fed-batch phase. Concerning biomass production from fructose, the overall yield for pCB3 was equal to 0.32 ± 0.01 g_X_·g_S_^−1^ similar to the one obtained for pCB1. CO_2_ production from biomass was equal to 0.87 ± 0.02 g_CO2_·g_S_^−1^ for pCB3. Like for pCB1, biomass overall yield was decreased in the benefit of CO_2_ production.

No organic acids were produced during the experiment. There was a transient pyruvate production after the beginning of the fed-batch phase with 9 g for pCB3, which was quickly consumed. The overall biomass production from nitrogen yield 5.21 ± 0.41 g_X_·g_NH3_^−1^ for pCB3 was comparable to pCB1. This difference appears non-significant based on the value of the standard deviation. Experimental data presented a 6 to 12.5% decrease compared to the theoretical biomass production yield from ammonium (5.96 ± 0.41 g_X_·g_NH3_^−1^).

During batch phase, the maximum growth rate reached was 0.20 ± 0.01 h^−1^ for Re2133/pCB3 and 0.24 ± 0.01 h^−1^ for Re2133/pCB1 (Fig. 4d, f; Table 1). During the fed-batch phase, the specific growth rate applied was 0.04 ± 0.01 h^−1^ for pCB1 and 0.03 ± 0.01 h^−1^ for pCB3. There was no significant difference in the fed-batch monitoring strategy based on the growth rate imposed by the nitrogen feeding. For both strains, fructose and ammonium consumption rates were close during fed-batch phases.

For pCB3, the respiratory quotient was stable around 1.14 ± 0.02, all along the fed-batch phase (Fig. 4g, i; Table 1). Specific CO_2_ production and O_2_ consumption rates were stable around 2.10 ± 0.40 and 1.84 ± 0.30 mmol·g_X_^−1^·h^−1^, respectively.

#### Single-cell analysis

In all culture conditions, an increase in permeabilized cells percentage was observed just after the beginning of the fed-batch phase (Fig. 5a, c). This could be explained by the transient nitrogen depletion at the end of the batch phase. During the fed-batch phase, the percentage of permeabilized cells kept on increasing for pCB3 up to 13%, while the value remained stable around 3% for pCB1. During fed-batch phase, relative extracellular fluorescence in the supernatant was stable around 200 RFU for pCB1 and it increased continuously from 200 to 470 RFU for pCB3 and seemed to be correlated to permeability increase. However, the last two points for Re2133/pCB3 have to be considered with caution as optical density decreased because of cell lysis, as confirmed in cytograms (*data not shown*).

A comparison of plasmid expression level between our reference strain Re2133/pCB1 and Re2133/pCB3 strain carrying the PSK system was carried out. Regarding flow cytometry distributions (Figs. 5d, f), both strains presented a similar fluorescence intensity distribution that was normal and stable through batch phase. At the beginning of the fed-batch, boxplots distribution ranges for pCB1 were significantly more disrupted than with pCB3. After 60 h culture up to the end, boxplot distribution ranges for pCB3 were wider in direction of the first quartile. This was consistent with the increased relative extracellular fluorescence intensity observed for pCB3 beforehand, as permeable cells presented lower fluorescence intensity (*data not shown*). Therefore, higher permeabilization percentage led to increased eGFP leakage, to higher relative extracellular fluorescence intensity, and to wider boxplots distribution ranges.

The decimal reduction rates measured by plate count and flow cytometry data (Fig. 5g, i**)** for both pCB1 and for pCB3 through time were not significant and no plasmid loss was detected.

## Discussion

The aim of this work was to assess robustness of engineered strains under nitrogen-limited fed-batch cultures with *C. necator*. Strain robustness was studied under two sugar feeding strategies (pulses and continuous) and with two plasmid stabilization systems (kanamycin resistance and Post-Segregational Killing *hok/sok*). Strain robustness was defined as the ability of cells to maintain homogenous growth, production performances and plasmid expression levels, among individuals over a long period of culture and was evaluated via plasmid expression stability, cultivability, membrane integrity and macroscopic cell behavior.

Traditionally, substrate feeding strategy for fed batches can be carried out either by continuous feeding requiring programmable pumps or easily by pulse addition. In previous works, the two strategies were applied with *C. necator*, the pulse-based strategy for fructose feeding was applied for the production of isopropanol (Marc et al. 2017) and the continuous strategy (maintain at 20 g·L^−1^) was led during alka(e)ne production (Crepin et al. 2016); both in nitrogen limited fed-batch mode. It is well-known that sugar-pulsed strategy led to fluctuating environments, which may reduce process performance and strain robustness, as reported in some studies (Hewitt et al. 2007; Lara et al. 2006; Limberg et al. 2017). In *E. coli*, high glucose pulses might be accompanied by an overflow metabolism in strict aerobic conditions, which might lead to acetate production (Lara et al. 2009; Neubauer and Junne 2010). High substrate concentrations in the feeding zone during pulses might also lead to enhanced respiratory activity and so, to dissolved oxygen depletion (Lara et al. 2006). In this case, under oxygen limitation, a fermentative metabolism might occur and divert carbon flow toward acetate, formate and ethanol productions (Lara et al. 2009; Neubauer and Junne 2010). If organic acids are produced in high enough concentrations, pH decrease might also occur locally in the feeding zone. All these phenomena might lead to strain robustness disruption. In this work, a continuous feeding strategy was applied during the culture of the strain Re2133/pCB1 under selective pressure and results were compared to pulse-feeding strategy, in terms of strain robustness.

No significant impact was observed on the specific growth rate or on overall production yields, as a result of the difference in the initial fructose concentration (30 *vs.* 50 g·L^−1^) during the batch phase (0.25 ± 0.01 h^−1^ for pulse feeding and 0.22 ± 0.01 h^−1^ for continuous feeding). During the fed-batch phase, the growth was dictated by the dynamics of the nitrogen-feeding conduct and was equal for both feeding strategies. The global biomass production yields on fructose were found higher in the continuously fructose feeding strategy. No organic acid was detected with continuous feeding, whereas organic acid production occurred in the pulse experiment, representing up to 15% of the consumed carbon. Therefore, organic acids and growth competed for the carbon. Pyruvate was transiently produced after nitrogen depletion and re-consumed for both fructose feeding strategies. This transient phenomenon might likely be due to a carbon overflow at the onset of nitrogen limitation as previously reported in engineered *C. necator* strains (Crepin et al. 2016; Marc et al. 2017). The explanations for the production of citrate, acetoacetate and acetate are given as follows. First, the production of citric acid was reported during fed-batch cultures with alka(e)ne engineered *C. necator* strains (Crepin et al. 2016). However, quantities measured during alka(e)ne production were lower (1 g·L^−1^ on fructose; 2 g·L^−1^ on CO_2_) than the ones determined in this work with pulse feeding (max. 20 g, corresponding to 6.7 g·L^−1^). The accumulation of citrate could be explained by a disruption in the TCA cycle via the inhibition of aconitase. Nitrogen limitation is known to inhibit aconitase’s activity in some oleaginous microorganisms, leading to citrate excretion (Evans and Ratledge 1984; Ratledge 2002). As a natural producer of PHB, *C. necator* could, to some extent, follow the patterns of metabolic regulation of oleaginous microorganisms, which might be favored under alka(e)ne production or with fructose excess under nitrogen-limited culture conditions. Second, the accumulation of acetoacetate during fed-batch phase might be due to the redirection of the carbon flow from acetyl-CoA toward the biosynthesis of PHB. As this pathway has been deleted in the *C. necator* Re2133 strain downstream of the acetoacetyl-coA, aceto-acetate was likely produced by the acetoacetyl-CoA transferase that is naturally present in *C. necator* (Grousseau et al. 2014). Third, the accumulation of acetate coming from the conversion of the acetyl-CoA via phosphate acetyltransferase—acetate kinase pathway has already been reported in the PHB–deleted strains and engineered strains (Crepin et al. 2016) or in response to oxygen limitation (Tang et al. 2020).

*C. necator* presents a natural carbon overflow metabolism toward the production of PHB in conditions of nutritional limitation (*e.g.* nitrogen, phosphate), which can represent up to 80% of its dry cell mass (Ryu et al. 1997). However, as said above, the PHB biosynthesis pathway was deleted in our strain. One might hypothesize that with fructose pulses under sugar fluctuating conditions, the transitory excess carbon flow was re-directed toward the production of organic acids. In Marc et al. (2017), no organic acids were produced during the same nitrogen-limited phase with fructose pulses. Therefore, in isopropanol engineered strains, the redirection of this carbon overflow was probably totally drawn by the isopropanol biosynthetic pathway. This was not the case for alka(e)ne production in *C. necator*, where the amount of organic acids produced during fed-batch increased with continuous fructose feeding (Crepin et al. 2016). The efficiency of the carbon flow redirection logically depends on the design of the synthetic pathway.

The percentage of PI-permeabilized cells was globally higher during fructose pulse feeding. This increase in cell permeability was correlated with higher relative extracellular fluorescence intensity, due to higher eGFP leakage outside the cells. The main effect of higher sugar concentrations on microbes is osmotic shock, since water diffuses through membranes as a response of increased osmotic pressure. Therefore, water activity decreases in cells (Lengeler 1998; Parish 2006). As a result, enzyme activity might be disrupted, which could lead to a weakening of DNA structure, growth inhibition or cell permeabilization (Parish 2006). The response toward high sugar concentrations highly depends on the microorganism (Kushner 1964). A promoter inducible by carbon starvation (*csiE*) has been used to control GFP expression in *E. coli* (Delvigne et al. 2011a,b) to study the impact of sugar-mixing imperfections on protein expression and/or excretion. Three cultivation modes were investigated: chemostat, fed-batch and scale down reactor (*abbr.* SDR, that mimics heterogeneity in large scale bioreactors through a recycle loop). GFP excretion in the medium was dependent on the nature of stress encountered by cells in a given bioreactor conduct. During strict sugar limitation under prolonged culture conditions in chemostat and fed-batch modes, permeabilized cell percentage was higher and was correlated to higher GFP leakage intensity. Under sugar fluctuating environments, in chemostat (switch from batch to chemostat, or abrupt changes in dilution rate) and in SDR (glucose gradient in recycle loop), GFP excretion was slowed down. Indeed, such complex extracellular fluctuations (*i.e.* transitory glucose non-limiting conditions) might have induced an overflow metabolism and inactivated the carbon-limitation promoter (*csiE*). In this case, membrane was in a better state (*i.e.* low permeability) and this could be due to an adaptation of cells to constantly fluctuating environments in SDR. GFP leakage was shown to be correlated with higher permeabilization percentages, and so, was lower for SDR compared to fed-batch mode. However, our results were different concerning the impact of environmental fluctuations on cell permeability, as cells tended to be more permeable under pulse feeding compared to continuous feeding. First, the intensity of sugar concentration fluctuations was different, as fructose concentrations varied from 20 to 50 g·L^−1^ in our work, and glucose concentrations from 0 to about 1 g·L^−1^ in Delvigne et al. (2011a, b). Then, fluctuations occurred more regularly in the SDR (every 8 min) than in our fed-batch culture (12–18 h in between pulses), which might favor the adaptation of cells to fluctuating environments in SDR. Finally, cultures conditions were glucose-limited in Delvigne et al. (2011a, b), which was not the case in our work ([fructose] > 20 g·L^−1^). Glucose-limitation led to a drop of cell viability (*i.e*. increase in cell permeability, by PI-staining) in chemostat, whereas cells adapted to fluctuating culture conditions in SDR.

The decimal reduction rate was considered not significant for continuous feeding when calculated by plate count and flow cytometry. For pulse feeding, decimal reduction rates were low (< 0.2), except after the second fructose pulse (0.3 at 55 h by flow cytometry) and the third one (0.7 at 70 h by plate count), indicating a slight plasmid stability loss. In both culture conditions, the majority of cells were plasmid-expressing cells. But they presented heterogeneous plasmid expression levels either after fructose pulses or after extended culture durations under selection pressure. Until 30 h for pulses and 50 h for continuous feeding, plasmid expression level distribution could be considered close to normal, according to boxplot representation. However, after these moments, expression level distributions were noisy until the end of fed-batch phase, because of two phenomena. Firstly, in both cultures, increased cell permeability was correlated with increased eGFP leakage, which contributed to widen fluorescence intensity distribution, as permeabilized cells presented globally a lower fluorescence intensity. Secondly, during pulse feeding a slight increase in decimal reduction rate was detected (at 55 and 70 h) and might reveal a slight loss in plasmid expression level (*i.e.* widening of the first quartile) that could impact fluorescence intensity distribution.

The continuous fructose feeding strategy which allowed homogeneous and stable culture conditions during fed-batch phase was selected to pursue strain robustness evaluation. The impact of two plasmid stabilization mechanisms on strain robustness was studied: kanamycin resistance and Post-Segregational Killing (PSK) *hok/sok*. The reference culture (*i.e.* Re2133/pCB1 without kanamycin addition) and the cultures led under plasmid stabilization conditions (*i.e.* Re2133/pCB1 with kanamycin and pCB3 with PSK system) were compared on their impact on macroscopic behavior, plasmid expression levels and cell physiology.

Both plasmid stabilization systems presented close biomass and CO_2_ production yields from fructose between the two stabilization systems. However, there was a significant reduction in the biomass production yield from fructose compared to the theoretical value. Indeed, overall biomass production yield from fructose was 40% lower compared to reference value (0.53 g_X_·g_S_^−1^, Aragao et al*.* 1996) and the missing carbon was deviated toward the production of CO_2_. It is likely due to the presence of the plasmid in host cells and to the production of eGFP by plasmid-expressing cells.

During fed-batch phase, the permeabilized cell percentage decreased and stabilized around 5% for pCB1 without antibiotic. However, it increased up to 10% for pCB1 with kanamycin and up to 15% for pCB3. As a result, the strains reached different relative extracellular fluorescence intensity, from lowest to highest: pCB1, pCB1 with kanamycin and pCB3. It appears that stabilization systems (kanamycin resistance and PSK system) led to an increase in eGFP leakage outside of cells. The distribution profiles of fluorescence at the single-cell level widen at the end of culture (*i.e.* stably for pCB3 and noisy for pCB1 + kanamycin), when relative extracellular fluorescence intensity and percentage of permeabilization increased, for plasmid stabilization systems. Plasmid expression levels were more stable throughout culture for the strain Re2133/pCB3, especially at the beginning of the fed-batch phase compared to the strain Re2133 with and without kanamycin. Therefore, Re2133/pCB3 presented an advantage in terms of strain robustness compared to the strain Re2133/pCB1 all along the culture, based on more homogeneous plasmid expression levels at nitrogen depletion and until 60 h of culture. Maintaining plasmid expression levels without the use of antibiotics (with PSK *hok/sok*) might be interesting to avoid their addition in cultures at industrial scale, to decrease the economic cost of the bioprocess and the risk of multidrug resistance issue. We can precise that the intracellular fluorescence intensity distribution and the levels of extracellular fluorescence intensity in the medium (eGFP leakage) reached in this study were far lower than the values reached in our previous work (Boy et al. 2020) with the strain Re2133/pKRSF1010-P_j5_-eGFP in flasks (*i.e.* eGFP constitutively induced by the strong promoter P_j5_). Thus, eGFP leakage in this work was not due to a too high intracellular fluorescence intensity that the host cells would not be able to cope with.

For all fermentation conditions, plate count and flow cytometry measurements gave comparable cell number for the total cell population and the plasmid expressing cells population. So, neither of the plasmid stabilization system studied impacted cell cultivability. The decimal reduction rates calculated from flow cytometry and plate count data were not significant for both plasmid stabilization strategies, as it was already the case without selection pressure for Re2133/pCB1.

Due to its stability under well-controlled intensive culture condition, the plasmid pCB3 would be a valuable backbone to evaluate plasmid expression levels in new recombinant protein production conditions or for expressing novel biosynthetic pathways. Therefore, any modification in plasmid expression levels might be attributed to the recombinant protein production.

In conclusion, we demonstrated that the sugar feeding strategy for fed-batch mode is important to consider as it can have none negligible impact on microbial behavior and therefore on bioprocess performances. In nitrogen limited fed-batch cultures, a smooth continuous fructose feeding allowed a better carbon use for protein synthesis (avoiding organic acid excretion), a lower heterogeneity of the plasmid expression and a lower cell permeabilization. Indeed, it appeared clearly that pulsed base strategy was more stressful for the cells at the single-cell level, leading to a direct impact on the biomass production and thus on bioprocess performances. Among the stabilization systems tested here, the PSK system, an autonomous system independent on external addition of compounds showed the best ability to maintain plasmid expression level stability insuring a greater population homogeneity in the culture. Surprisingly, the kanamycin resistance system in presence of kanamycin, showed negative impact on plasmid expression level, growth and cell permeability in comparison with the culture of the same strain but without kanamycin addition. Therefore, in the case of engineered *C. necator*, the PSK system *hok/sok* appeared to be a relevant and an efficient alternative to antibiotic resistance system for selection pressure especially in the case of bioprocess development for economic and environmental reasons. The so-designed plasmid pCB3 would be an interesting tool to study plasmid expression levels for the production of other recombinant proteins or for expressing biosynthetic pathways.

## Data Availability

All data generated or analyzed during this study are included in the present work.
